# Design of a Dual-Technology Fusion Sensor Chip with a Ring Electrode for Biosensing Application

**DOI:** 10.3390/mi10020153

**Published:** 2019-02-23

**Authors:** Cheng Ma, Jin Zhu, Xiaolong Li, Wei Zheng

**Affiliations:** 1 College of Electronic and Information, Jiangsu University of Science and Technology, Zhenjiang 212003, China; 162210304221@stu.just.edu.cn (C.M.); lixiaolong@just.edu.cn (X.L.); zhengwei@just.edu.cn (W.Z.); 2 Zhenjiang Laboratory of Information Sensing and Transmission Technology for Smart Ocean, Zhenjiang 212000, China

**Keywords:** gold nanoparticles, quartz crystal microbalance (QCM), local surface plasmon resonance (LSPR), gold electrode, dual-technology chip

## Abstract

Quartz crystal microbalance (QCM) is still a new high-precision surface detection technique. However, the adsorption quality detected by the QCM currently contains a solvent-coupling quality and cannot separate the actual biomolecular mass. Local surface plasmon resonance (LSPR) can detect the mass of biomolecules, but requires a certain contrast between the solvent of the surrounding medium and the refractive index of the adsorbed layer. The sensor chip, combining two compatible technologies, can realize the simultaneous detection of biomolecules and improve the refractive index sensitivity. The structure of our chip is to prepare the ring-shaped gold electrode on the upper surface of the quartz crystal, the circular gold electrode on the bottom surface, and the spherical gold nanoparticles arrays in the center region of the ring electrode to form a QCM/LSPR dual-technology chip. Through simulation, we finally get the size of the best energy trap by the two electrodes on the upper surface and the lower surface: the ring-top electrode with a thickness of 100 nm, an inner diameter of 4 mm, and an outer diameter of 8 mm; and the bottom electrode with a thickness of 100 nm and a radius of 6 mm. By comparing the refractive index sensitivity, we chose a spherical gold nanoparticle with a radius of 30 nm and a refractive sensitivity of 61.34 nm/RIU to design the LSPR sensor chip.

## 1. Introduction

With the development of science and technology, the research of surface probe technology is deepening. The use of single technology is not enough to obtain the parameters of the complex biofilm structure. Therefore, combining the two technologies can expand the amount of information detected by the system and improve the reliability of the detection information and precision. Quartz crystals have high sensitivity, small size and strong vibration resistance, so they can be used to measure the parameters, which other instruments with low precision cannot measure [1]. The principle of quartz crystal microbalance (QCM) is to use the piezoelectric effect [2,3,4] of quartz crystal to convert the subtle mass change on the quartz crystal into a change in the vibration frequency. The detection accuracy of QCM is proportional to the resonant frequency. QCM was originally applied to air or a vacuum to detect substances adsorbed on its surface. With the development of science and technology, QCM has progressed to have more applications in liquid [5,6,7,8,9]. However, QCM is only suitable for rigid films that are thin enough and evenly spread relative to quartz crystals. Quartz crystal microbalance with dissipation monitoring (QCM-D) can determine whether the surface adsorbs soft/viscoelastic materials or rigid material by observing the change of dissipation value, Δ*D*, and can also monitor the viscoelasticity and structural changes of the material in real time. However, because the quality of QCM-D detection included the coupling quality of the solvent, the quality of the actual biomolecule “dry mass” cannot be measured [10]. Local surface plasmon resonance (LSPR) is an optical detection technology with high sensitivity and real-time detection of binding between biomolecules. When light waves having the same electron frequency as the metal nanostructures are irradiated onto the metal surface, the charge on the metal surface oscillates, the electromagnetic field is enhanced, and the refractive index change in the vicinity of the metal nanostructure becomes sensitive, so that the sensitivity is improved [11]. In liquid, LSPR measures the “dry mass” of the biomolecules but requires different refractive index between the adsorbed layer and the solvent. The structural properties of the adsorbed layer cannot be obtained by LSPR alone. With the rapid development of QCM’s sensing technology, QCM-based sensors have been widely used in applications such as the detection of chemicals, biomolecules, and bacteria. Applying different materials to the surface of QCM allows it to be used for non-enzymatic glucose detection [12], determination of protein concentration [13], determination of prostate-specific antigen (PSA), and detection of PSA-α1-antichymotrypsin (ACT) complex (75%) in human serum [14]. The QCM immunosensor can also be used to detect epidermal growth factor receptor (EGFR) [15,16] and the like. When LSPR is excited, both absorption and scattering are greatly enhanced. Therefore, spectroscopy is the simplest method of detecting LSPR on metal nanostructures and is typically based on extinction, or scatterometry, measurements. Extinction is commonly used to characterize systems containing large amounts of nanostructures, such as nanoparticle colloids. The extremely intense and highly restricted electromagnetic field caused by LSPR provides very sensitive probes to detect small changes in the dielectric environment around the nanostructures, which is particularly attractive for sensing applications [17,18].

If we run both technologies on the same chip at the same time, we can combine the advantages of both technologies. By complementing the advantages of the two technologies, it is possible to obtain data that cannot be obtained by a single measurement technique. QCM-D can measure the total mass of the adsorbed molecular film (including the coupling solvent mass). LSPR can measure the actual adsorbed molecular mass, and we can get the coupling solvent quality by subtracting the molecular mass from the total mass. Both the gold electrode on top of the traditional QCM-D sensor and the gold nanodisc have a layer of silicon dioxide spacer. Using the principle of light reflection, the SiO_2_ spacer layer through which the light passes is then reflected by the gold electrode at the top, so that the light is twice irradiated to the gold nanodisc. Traditional QCM-D sensors are currently produced and commercialized. Hao et al. [19] have proposed to fabricate a 400 μm diameter window in the center of a circular gold electrode under a conventional QCM device, and to prepare a 400 μm × 400 μm array of Au LSPR nanodisc in the center of the upper circular gold electrode. Using the light transmission principle to illuminate the Au LSPR nanodisc, Ferhan et al. [20] have combined traditional QCM-D with local surface plasmon (LSPR) and studied a combined measurement method. For better research, they have integrated the reflective mode LSPR sensor with the traditional QCM-D instrument platform and used a titanium oxide-coated sensing substrate to study vesicle adsorption and amphipathicity.

The structure of the chip we designed was compared with the conventional QCM-D device, and the top circular gold electrode was replaced with a ring-shaped gold electrode, and the optimal ring gold electrode size was obtained by ANSYS software simulation. Using a pipette to take a small amount of nanosolution titrated in the center of the ring-shaped gold electrode is equivalent to directly preparing on the quartz crystal, allowing it to dry naturally at room temperature. Finally, gold nanoparticles with narrow distribution are adsorbed on the surface of the quartz crystal. The preparation of spherical nanoparticles by a conventional method is simple, and they were directly placed on a quartz wafer without large influence on the electrode structure and the conductivity of the electrodes. The top gold electrode is ring-shaped, and the fundamental frequency of the gold electrode quartz crystal changes by Δ*f* = 8574.3 Hz, which is completely negligible compared with the chip’s fundamental frequency *fs* = 4.98 MHz. In terms of LSPR, our LSPR spectrum measurement works in reflection mode. The light is vertically irradiated onto the light-sensitive area composed of the gold nanospheres, and the light is irradiated onto the gold electrode through the quartz crystal and is vertically reflected back, so that the light is twice irradiated to the gold nanodisc array. The nanoparticle undergoes two LSPR spectral absorptions by one reflection and two transmissions, ultimately enhancing the spectral intensity.

## 2. Materials and Methods

### 2.1. Theory

QCM is a kind of high-precision measuring instrument. The quartz crystal sensor uses AT-cut oscillator [21] with metal electrode prepared on both sides of the quartz crystal. After the electrodes are connected to the wires, the chips are encapsulated to form the quartz crystal resonator. As early as 1959, German physicist Gunter Sauerbrey discovered and pointed out that the decline of the crystal’s resonant frequency was proportional to the mass of the surface attachment [22]. However, it only worked in air or vacuum environments, and in liquids, the Sauerbrey equation cannot be established because the viscosity of the liquid dissipates energy. In 1996, Rodahl [23] proposed equations for the change of liquid phase dissipative factor (Δ*D*):

(1) $$\Delta D=2{\left(\frac{f_{0}}{n}\right)}^{1/2}{\left(\frac{\varepsilon Q_{1}}{\pi \mu Q_{q}}\right)}^{1/2}.$$

Unlike QCM, QCM-D could measure both the change of quartz crystal frequency and the change of the dissipation value. QCM-D obtains frequency change (Δ*f*) by monitoring the change in molecular mass adsorbed on the sensor, such as the Sauerbrey formula:

Δ*f* = −2.26 × 10^−6^*f*^2^ Δ*m*/*A*, (2)

and the dissipative value change Δ*D* is obtained by the viscoelasticity of the adsorption mass, according to the attenuation formula

*A*(*t*) = *A*_0_ · *exp*(−*t*/*τ*) · *sin*(2*πft* + *φ*), (3)

*D* = 1/*πft*. (4)

Compared with SPR sensor technology, the LSPR technique uses noble metal nanoparticles with special optical properties as the carrier of the sensitive membrane while retaining SPR technology, which makes the sensor device simple and more sensitive to the medium environment around the nanoparticles [24,25]. LSPR is an optical phenomenon. When the incident light is irradiated on the surface of precious metal particles, the coupling of the noble metal nanoparticles produces a plasma resonance that produces one or more maximum values on the extinction efficiency distribution curve [26,27]. The extinction efficiency is related to the size and shape of the nanoparticles [28,29].

We designed a photoelectric sensor, which combines QCM-D and LSPR technology to achieve complementary advantages. QCM-D provides a lot of molecular and coupling solvents for the sensor. The environment meets the requirement for LSPR to measure the mass of biomolecules in liquid and make up for the shortcomings of QCM-D detection quality, including the coupling quality of the solvent.

### 2.2. Structure

**Figure 1:**
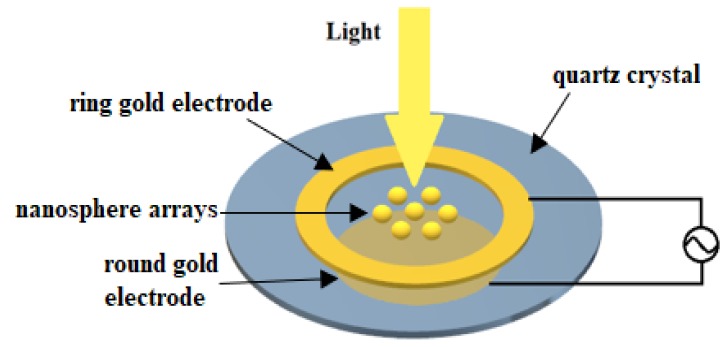
Structure of quartz crystal microbalance (QCM)/local surface plasmon resonance (LSPR) chip.

The QCM/LSPR dual-technology chip integrates reflectance mode LSPR into a QCM, which has a ring-shaped gold electrode. The ring-shaped gold electrode is prepared as the top electrode, and a circular gold electrode is prepared as the bottom electrode. An alternating current (AC) voltage of 5 V is applied on the upper and lower electrodes to resonate the crystal. The chip structure is shown in Figure 1. The paper obtains the best electrode size by finite element analysis [30]. Gold nanoparticles were synthesized by reduction of HAuCl_4_ solution with sodium citrate solution. Nanoparticles were immobilized by drop-evaporation from the aqueous synthesized solution. The droplet was placed on the quartz in the inner region of the ring electrode and dried at ambient conditions. This approach allowed for the investigation of the LSPR spectrum, which was scattered by nanoparticles. In order to improve the uniformity of the nanoparticle size and the reproducibility of the experiment, a second method can be employed in later experiments to combine the gold nanoparticles with QCM. First, 40 mL of 1.40 mM HAuCl_4_ (mM = mmol/L) and 400 μL of 0.213 M cysteamine hydrochloride were mixed in a glass vial with a capacity of 100 mL. The mixture was then stirred at room temperature and under dark conditions for 20 min. After 20 min, 10 mL of freshly prepared 10 mM NaBH was quickly added to the above mixture, and then regenerated and stirred for 30 min to obtain positively charged gold nanoparticles stabilized by cysteamine molecules [31]. Niidome et al. obtained gold nanoparticles AuNPsCH(+) stabilized by cytoamine [32]. After obtaining AuNPsCH(+), the suspension with a concentration equal to 5 mg·L^−1^ was sent to the surface of the QCM chip at a steady flow rate of 0.08 mL·min^−1^ (1.33 × 10^−3^ cm^3^·s^−1^) by a microfluidic pump. The AuNPsCH(+) in the solution was deposited for 10 to 300 min [33].

Since the gold nanoparticles have stable chemical properties and high optical sensitivity, the gold nanoparticle arrays were placed in the central region of the ring-shaped gold electrode [34,35,36,37]. When the beam is directed at the center of the ring electrode, the nanoparticles absorb the beam for the first time. Then, the light beam is transmitted through the quartz crystal and irradiated to the gold electrode at the bottom. The light beam is irradiated onto the gold nanoparticle by the reflection of the bottom gold electrode so that the nanoparticle absorbs the light beam for the second time, thereby enhancing the absorption spectral intensity and improving the optical sensitivity. The shape and size of nanoparticles in gold nanoarrays affect the absorption peak of the spectrum. In this paper, the spherical gold nanoparticles are selected to design the LSPR sensor chip.

## 3. Procedures

### 3.1. Electrode Size

We used the finite element analysis method to find the electrode size of the best energy-trapping graph by ANSYS software. QCM consists of a ring-shaped gold electrode above a quartz crystal and a circular gold electrode at the bottom. The inner and outer radius and thickness of the ring-shaped gold electrode, as well as the radius and thickness of the circular gold electrode, have a direct impact on the performance of QCM. We chose the electrodes with a thickness of 100 nm and a quartz crystal with a thickness of 333 μm to study the effect of the size of the electrode radius on the resonant frequency of QCM [38].

**Figure 2:**
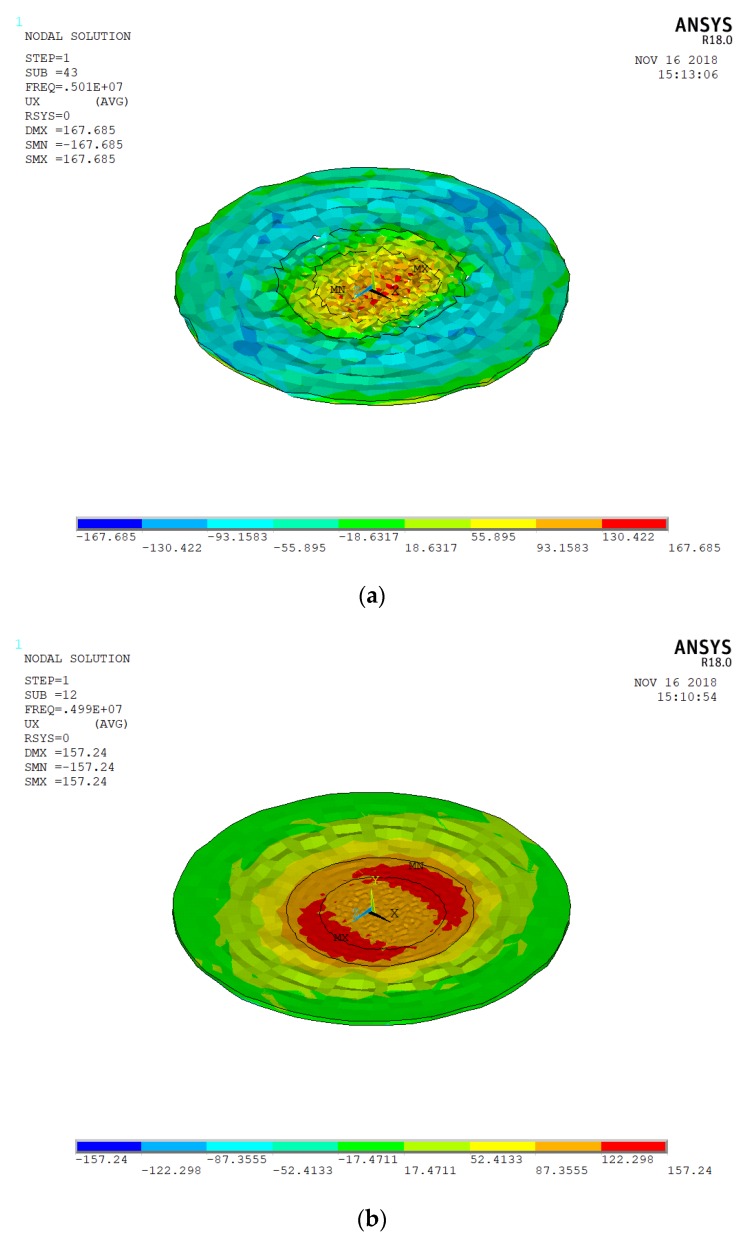
(**a**) The energy-trapping diagram of the 43rd modal subsets; (**b**) the energy-trapping diagram of the 12th modal subset.

**Figure 3:**
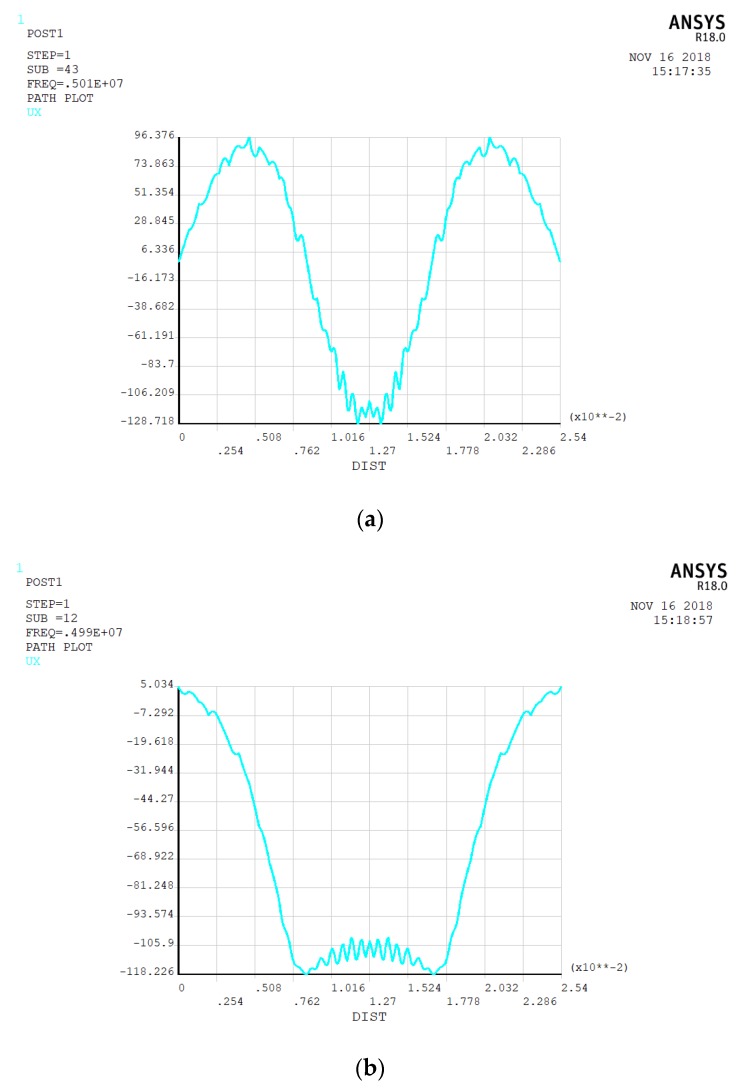
(**a**) The vibration displacement curve of the chip along the radius corresponds to Figure 2a; (**b**) the vibration displacement curve of the chip along the radius corresponds to Figure 2b.

For the top gold electrode, we first select the ring-shaped electrode with an inner radius of 4 mm and an outer radius of 6 mm to obtain the best energy-trapping diagram and corresponding vibration displacement curve. Figure 2 is an energy-trapping diagram of two subsets extracted from the simulation results of vibration mode. Figure 3 shows their vibrational displacement curves along the corresponding radius.

In Figure 2a, the energy of harmonics transmits to the non-electrode region, and the trapping effect is not obvious in Figure 2a. Figure 2b is more consistent with the best trap effect, and the maximum energy is confined to the central area of the electrode. Thus, the chip resonance is at 4.989680 MHz with single oscillation frequency. Figure 3a shows other vibrational modes of the quartz crystal. Since this paper adopts a thickness shear mode, these vibrational modes do not meet the requirements. In Figure 3b, the vibrational displacement curve is generally a normal distribution curve. The displacement amplitude in the central region is relatively small, and the displacement amplitude of the electrode region is larger than that of the central region, but the difference between the two regions is not obvious, so the electrode size needs optimization.

**Figure 4:**
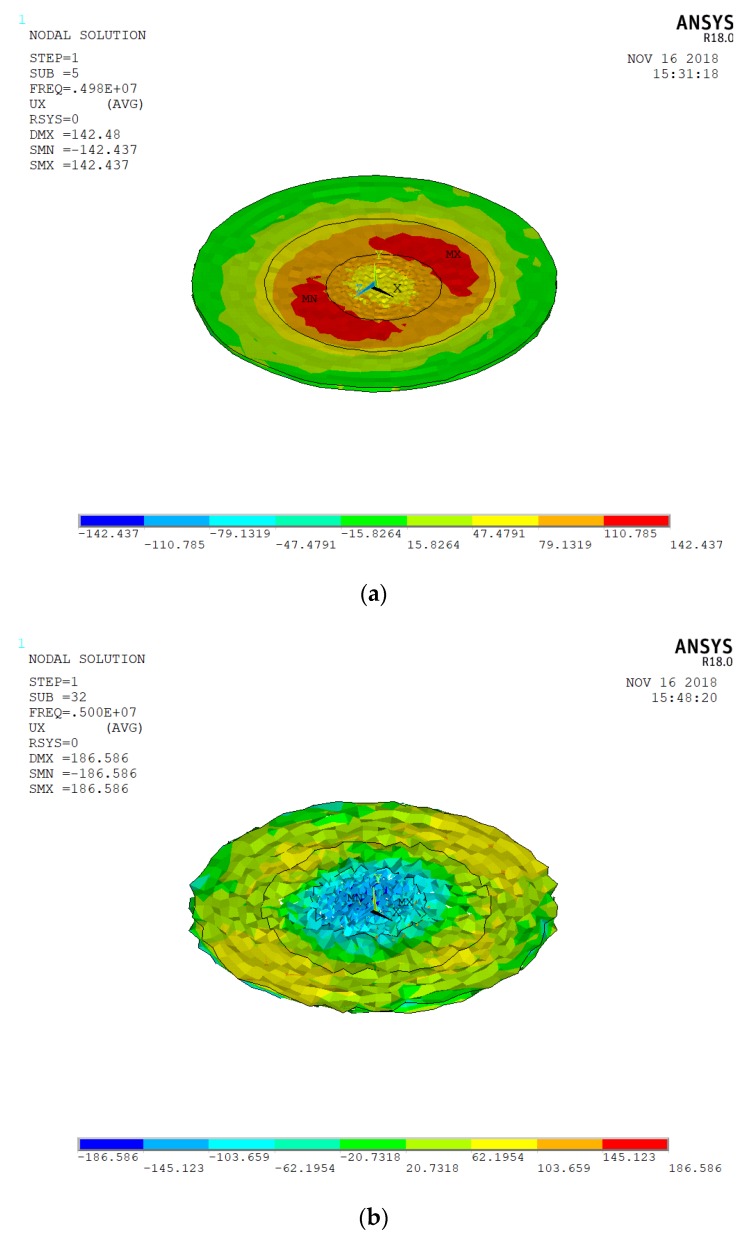
(**a**) The energy-trap graph corresponding to the external radius of 8 mm; (**b**) the energy-trap graph corresponding to the external radius of 10 mm.

**Figure 5:**
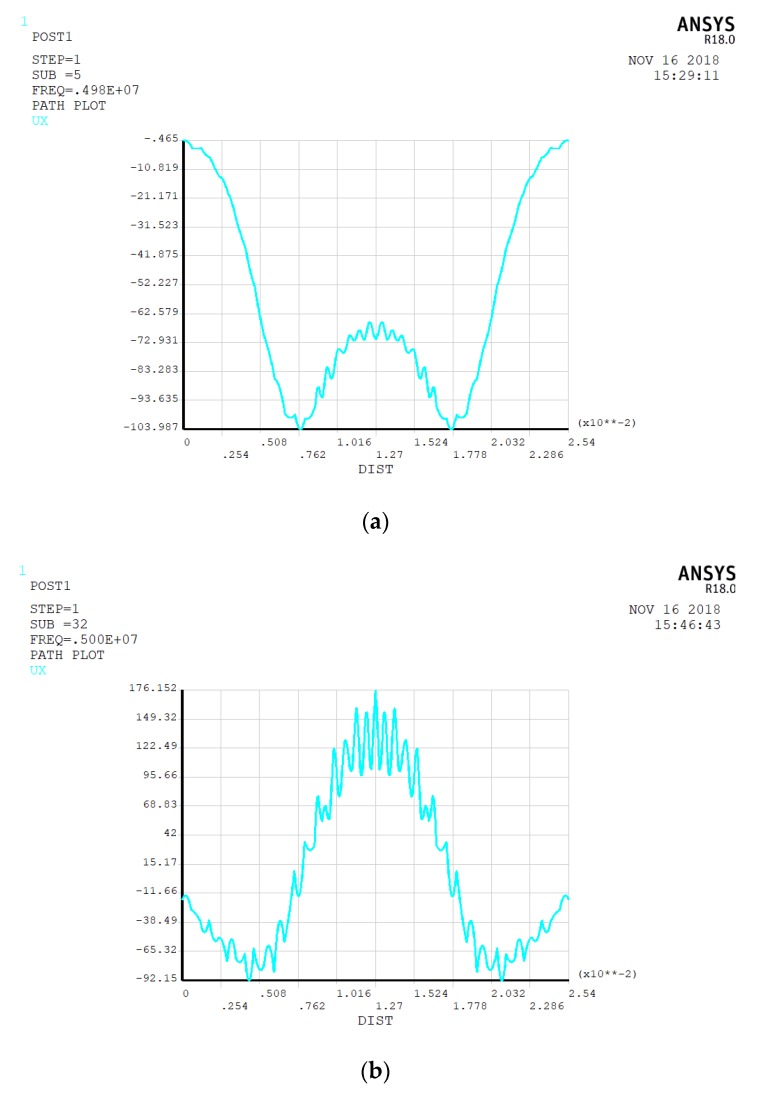
(**a**) The curve of vibration displacement at the outer diameter of 8 mm; (**b**) the curve of vibration displacement at the outer diameter of 10 mm.

**Figure 6:**
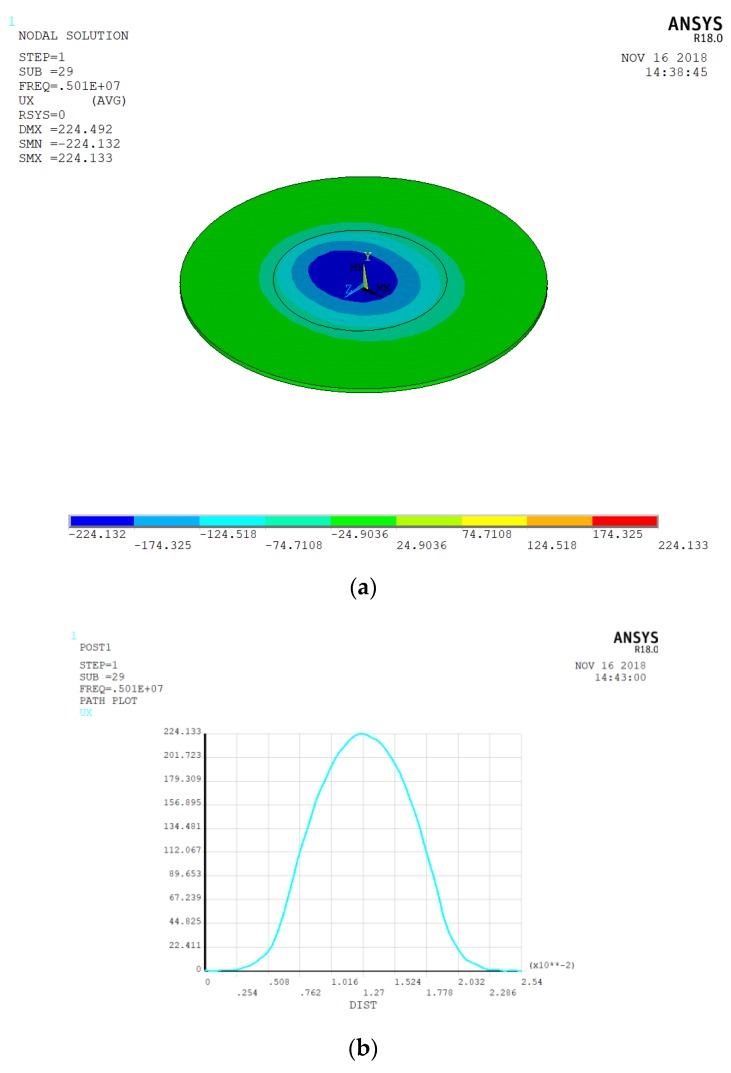
(**a**) The energy-trapping diagram of a circular gold electrode with a radius of 6 mm; (**b**) the vibration displacement curve corresponding to a 6 mm circular gold electrode.

We kept the ring-shaped gold electrode the same size, but the outer radius was increased to 8 and 10 mm. The energy-trap diagram is shown in Figure 4, and Figure 5 shows the corresponding vibration displacement curve. The electrode frequencies are 4,983,940 Hz and 4,998,130 Hz, respectively. We find that the optimal energy-trap graph is obtained when the outer diameter of the electrode is 8 mm. Figure 5a shows the smoothest vibration displacement curve when the outer diameter is 8 mm. Figure 5b shows that the interference of the non-resonant frequency signal is more obvious in the central region, which has great influence on the oscillation frequency. Hence, the optimal size for the ring-shaped gold electrode is with 4 mm inner diameter and an 8 mm outer diameter. We find that the larger the outer diameter of the electrode ring, the greater the frequency of the electrode. The optimal size of the bottom electrode is 6 mm, and we get the best energy-trapping diagram and corresponding vibration displacement curve in Figure 6.

Combined with the influence of electrode size on the resonant frequency and vibration mode, the optimal size of the quartz crystal with the upper electrode as the ring shape and the lower electrode as the circular shape is 4 mm for the inner ring, 8 mm for the outer ring of the upper electrode, and 6 mm for the lower electrode.

### 3.2. Spherical Nanoparticles

**Figure 7:**
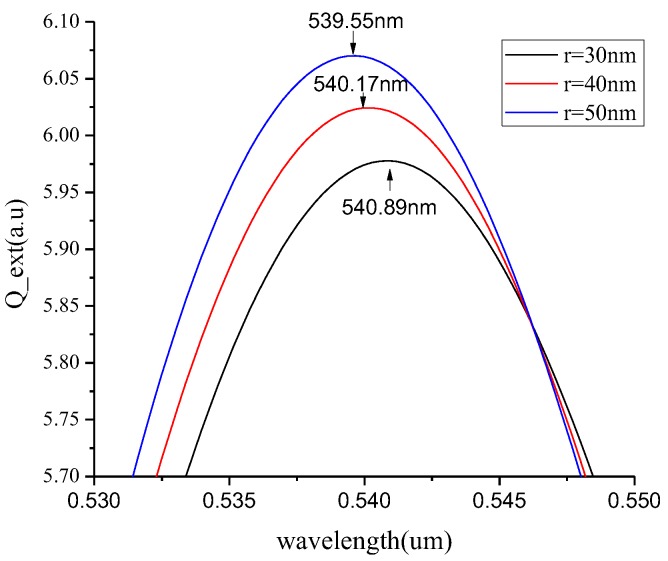
The absorption spectra of spherical gold nanoparticles of different sizes (30, 40, and 50 nm) with incident light wavelength of 530–550 nm.

We used the DDSCAT method [39,40,41] to study the absorption peaks and corresponding wavelengths of gold nanoparticles with different shapes and sizes in different media refractive indices. We control the same medium, which is benzene, under visible light of 530–550 nm. The absorption spectra of spherical gold nanoparticles of different sizes at the incident light wavelength of 530–550 nm are obtained, as shown in Figure 7.

**Figure 8:**
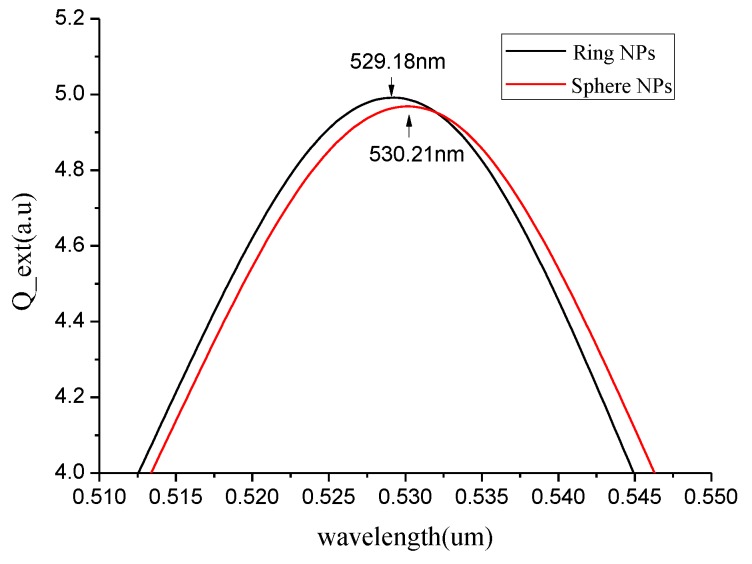
The absorption spectrum of ring nanoparticles and spherical nanoparticles at the wavelength of 510–550 nm.

It was obvious that with the increased radius of the spherical gold nanoparticles, the wavelength of the maximum absorption peak decreases. Moreover, obvious red shift occurs, and the maximum extinction efficiency was increasing. The absorption peak of 30 nm gold nanoparticles was 5.98 a.u., and the wavelength of the peak was 539.55 nm. Finally, we compare the absorption spectra of ring nanoparticles with an inner radius of 20 nm and an outer radius of 30 nm, and spherical nanoparticles with a radius of 30 nm in ethanol at an incident light wavelength of 510 to 550 nm, and the results are shown in Figure 8.

As shown in Figure 8, we know that the maximum extinction efficiency of the spherical nanoparticles is 4.992 a.u., and the corresponding wavelength is 529.18 nm. The maximum extinction efficiency of the ring nanoparticles is 4.968 a.u., and the corresponding wavelength is 530.21 nm. In the same volume, the ring nanoparticles were more efficient than spherical nanoparticles, but the corresponding wavelengths were smaller than spherical ones. In the case where the spherical radius and the outer annular radius were the same, the refractive sensitivity of the spherical nanoparticles is 61.34 nm/RIU, which is larger than the refractive sensitivity of the annular nanoparticles of 42.55 nm/RIU. Therefore, the LSPR sensor chip was designed by selecting spherical gold nanoparticles with a radius of 30 nm and a refractive index of 61.34 nm/RIU.

### 3.3. Effect of Spherical Nanoparticles on the Resonant Base Frequency of the Quartz Crystal ofGold Electrode

We add the spherical gold nanoparticle load to the gold electrode and obtain the energy-trap diagram and corresponding vibration displacement curve of the quartz crystal model loaded with mass load using ANSYS software. We obtain the fundamental frequency variation of gold electrode quartz crystal, Δ*f* = 8574.3 Hz, and find that compared with the fundamental frequency, *fs* = 4.98 MHz, it is completely negligible, so the spherical gold nanoparticles have no effect on the resonant fundamental frequency of the quartz crystal chip.

## 4. Results

Frequency shift and optical spectrum of dual-technology sensor chip in air and three different liquids were calculated. The related properties of three different liquids (water, ethyl alcohol, and benzene) are seen in Table 1. We controlled for the same shape and size of particles (selecting 30 nm gold nanosphere particles) and obtained the absorption spectra in the refractive index of different media.

**Figure 9:**
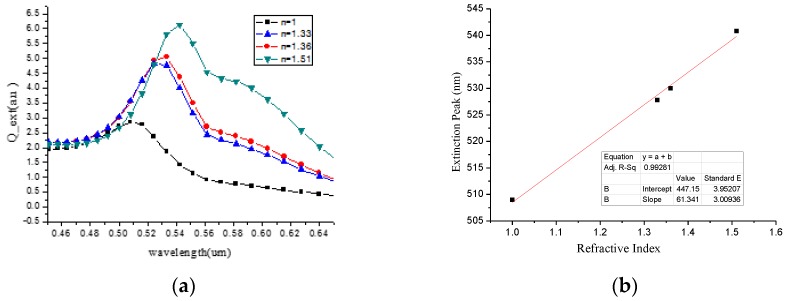
(**a**) The absorption spectra of the incident light at 450–650 nm and a radius of 30 nm in different media (1, 1.33, 1.36, and 1.51); (**b**) calculation of refractive index sensitivity for 30 nm nanoparticle array.

As Figure 9a shows, the maximum extinction efficiency increased with an increase of the refractive index of the liquid. The corresponding wavelength of absorption peak also increased with the increase of the refractive index of the liquid. The refractive index sensitivity was calculated by linear fitting in Figure 9b. The transverse axis is the refractive index of the four media, and the vertical axis is the corresponding peak position of each refractive index. The sensitivity of the refractive index is 61.34 nm/RIU. The refractive index of the media is 1, 1.33, 1.36, and 1.51 under visible light of 450–650 nm, and the results are shown in Figure 9b. The resonant frequency has obvious changes when the sensor is in different liquids. The resonant frequency has a shift from 4,983,940 to 4,964,690 Hz when *ε_r_* varies from 2.27 to 78.30.

## 5. Conclusions

Combining the above experimental results, we chose the ring gold electrode with an inner radius of 4 mm and an outer radius of 8 mm as the top electrode of quartz crystal, and a circular gold electrode with a radius of 6 mm as the bottom electrode. The vibration frequency of the chip is 4,983,940 Hz. The vibrational displacement curve of the circular gold electrode is a normal distribution chart, while the central region of the vibration displacement curve of the annular gold electrode is concave, which is obviously different from the electrode region. We selected the spherical gold nanoparticles with a radius of 30 nm and a refractive sensitivity of 61.34 nm/RIU to design the LSPR sensor chip. It is found, through experiments, that when the spherical gold nanoparticles are prepared in the middle of the ring-shaped gold electrode, there is no effect on the resonant base frequency of QCM. The purpose of the ring electrode is to make the central area transmit the light, which can be integrated in the structure of QCM and LSPR technology, forming dual-technology chips with ring electrode. The technique has good prospects for the molecular characterization of complex structures and high sensitivity for DNA and RNA detection.

## Figures and Tables

**Table 1:** QCM resonant frequency and frequency shift in different liquids.

Surrounding Medium	*n*	*ρ* (kg/m^3^)	*ε_r_*	*f* (Hz)	Δ*f* (Hz)
Air	1	1.29	1.00	4983940	0
Water	1.33	1000	78.30	4981470	2470
Ethyl alcohol	1.36	789	6.08	4977730	6210
Benzene	1.51	1880	2.27	4964690	19250

Liquid property: *n* is the refractive index sensitivity of the liquid. ***ρ*** is the density. *ε_r_* is the relative dielectric constant. *f* is resonant frequency of QCM in different medium. Δ*f* is the frequency shift in different liquid compared with those in air.
